# Association between Preoperative Medication Lists and Postoperative Hospital Length of Stay after Endoscopic Transsphenoidal Pituitary Surgery

**DOI:** 10.3390/jcm11195829

**Published:** 2022-09-30

**Authors:** Mary Saad, Benjamin Salze, Bernard Trillat, Olivier Corniou, Alexandre Vallée, Morgan Le Guen, Aurélien Latouche, Marc Fischler

**Affiliations:** 1 Department of Anaesthesia, Institut Curie, EPST, PSL Research University, 92210 Saint Cloud, France; 2 Institut Curie, INSERM, U900, PSL Research University, 92210 Saint Cloud, France; 3 Department of Anaesthesia, Hôpital Foch, 92150 Suresnes, France; 4 Department of Information Systems, Hôpital Foch, 92150 Suresnes, France; 5 Department of Medical Information, Hôpital Foch, 92150 Suresnes, France; 6 Department of Epidemiology-Data-Biostatistics, Delegation of Clinical Research and Innovation (DRCI), Hôpital Foch, 92150 Suresnes, France; 7 Faculty of Medicine, University Versailles-Saint-Quentin-en-Yvelines, 78000 Versailles, France; 8 Conservatoire National des Arts et Métiers, 75003 Paris, France

**Keywords:** pituitary adenoma, transsphenoidal surgery, length of stay, personal medication record, pharmaceutical preparations

## Abstract

Background: Endoscopic transsphenoidal surgery is the most common technique for the resection of pituitary adenoma. Data on factors associated with extended hospital stay after this surgery are limited. We aimed to characterize the relationship between preoperative medications and the risk of prolonged postoperative length of stay after this procedure. Methods: This single-center, retrospective cohort study included all adult patients scheduled for transsphenoidal pituitary surgery from 1 July 2016 to 31 December 2019. Anatomical Therapeutic Chemical codes were used to identify patients’ preoperative medications. The primary outcome was a prolonged postoperative hospital length of stay. Secondary outcomes included unplanned admission to the Intensive Care Unit, and in-hospital and one-year mortality. We developed a descriptive logistic model that included preoperative medications, obesity and age. Results: Median postoperative length of stay was 3 days for the 704 analyzed patients. Patients taking ATC-H drugs were at an increased risk of prolonged length of stay (OR 1.56, 95% CI 1.26–1.95, *p* < 0.001). No association was found between preoperative ATC-H medication and unplanned ICU admission or in-hospital mortality. Patients with multiple preoperative ATC-H medications had a significantly higher mean LOS (5.4 ± 7.6 days) and one-year mortality (*p* < 0.02). Conclusions: Clinicians should be aware of the possible vulnerability of patients taking systemic hormones preoperatively. Future studies should test this medication-based approach on endoscopic transsphenoidal pituitary surgery populations from different hospitals and countries.

## 1. Introduction

As hospitals are under increased pressure to decrease healthcare costs while maintaining quality of care, the identification of preoperative factors associated with increased postoperative healthcare has become highly relevant. Postoperative hospital length of stay (POLOS) is an automatable, global criterion and a reliable proxy to evaluate post-operative outcomes, quality of care and healthcare costs after elective surgery and, more specifically, after neurosurgery [1,2]. Although postoperative complications are associated with a prolonged postoperative length of stay (pPOLOS), much of the variance in regression models is attributable to preoperative characteristics in neurosurgical patients [3]. Several methods are used to assess the preoperative comorbidity burden. Diagnosis-based measures are based on subjective physician evaluation (e.g., ASA Physical Status Classification System) and/or patient self-reported comorbidities, sometimes requiring complex text-mining methods for data extraction. Medication-based methods evaluate the comorbidity burden through medication lists. While diagnosis-based comorbidity assessment methods have a high ability to predict mortality outcomes [4], medication-based methods are better correlated to healthcare utilization [5] such as POLOS [6] and hospital costs. The advantage of such medication-based approaches is that they are based on indisputable, objective, automatable and easily found preoperative variables, e.g., medication lists. Medication-based methods have been studied in the setting of general surgery [7] and various surgical specialties [8,9]. Endoscopic transsphenoidal hypophysectomy is the most common surgical approach for pituitary adenoma resection because it is associated with few complications and relatively short hospital stays [10,11]. However, data on factors associated with extended hospital stays after endoscopic transsphenoidal hypophysectomy are limited [12,13], and, to our knowledge, evaluation of the role of preoperative factors through medication-based models has not been tested. The objective of the present study was to characterize the association between preoperative medications, identified by their Anatomical Therapeutic Chemical (ATC) code, an international classification created by the World Health Organization Collaborating Centre for Drug Statistics Methodology (WHOCC), and pPOLOS after planned transsphenoidal hypophysectomy. We hypothesized that preoperative medications in patients undergoing endoscopic transsphenoidal hypophysectomy are associated with an increased risk of prolonged postoperative length of stay.

## 2. Materials and Methods

### 2.1. Ethical Approval

The study was approved by the Foch institutional review board (IRB: IRB00012437; approval number: 19-11-2) on 22 January 2020. The requirement for written informed consent was waived by the IRB as patients were informed that their medical data could be used for research purposes provided that their anonymity was respected upon admission to Foch Hospital. As required by the French Protection Act concerning the use of anonymized hospital data, we obtained permission to access this database from the French Commission on Information Technology and Liberties (Commission Nationale de l’Informatique et des Libertés, CNIL).

### 2.2. Cohort Selection

We conducted a retrospective descriptive analysis on all patients who underwent elective endoscopic transsphenoidal hypophysectomy at Foch Hospital, a tertiary academic hospital located in a Paris suburb in France. Patients included in the analysis were those who had surgery between 1 July 2016 and 31 December 2019. In case of multiple surgeries during the study period, we only included the index surgery. We excluded pediatric patients (aged less than 18 years), those with emergency surgeries (identified as those with no preoperative consultation and those with a consultation dating from more than 3 months as emergency surgeries have a specific consultation database not included in our extracted data) and those admitted to the ICU ahead of surgery. This study is in accordance with the Strengthening the Reporting of Observational Studies in Epidemiology (STROBE) reporting guidelines. The STROBE statement reporting checklist is provided in Table S1.

### 2.3. Data Source

Patients’ characteristics and preoperative medications were collected from Cesare™, a computerized software program for preoperative anesthetic evaluation (Bow Médical, Boves, France). Preoperative medications were defined using the Anatomical Therapeutic Chemical (ATC) classification system. In the ATC classification system, the active substances are divided into different groups according to the organ or system on which they act and their therapeutic, pharmacological and chemical properties. Drugs are classified into groups at five different levels. Cesare™ associates each medication trade name first with its corresponding International Non-Proprietary Name (INN), and then with its ATC code according to the ATC Classification System using the first four digits. In our analysis, we used the first level of the code, which indicates the anatomical main group and consists of one letter. There are 14 main groups. Each patient’s medical prescription was transformed into numerical variables that indicated the number of drugs in each level-1 ATC class that was present in the preoperative treatment. Surgical procedures were identified using the French classification of homogeneous patient groups (GHM) [14]. This classification, as well as most of the other medico-economic classifications used in the rest of the world, is derived from the Diagnosis-Related Groups (DRGs) classification system, developed in the 1970s in the United States [15] and based on the classification of hospital stays into a deliberately limited number of groups characterized by a double medical and economic homogeneity. Postoperative length of stay and hospital readmission were obtained using the hospital electronic health record system. Mortality data on the system were synchronized with France’s National Institute of Statistics and Economic Studies, Paris, France (INSEE) database, ensuring a near-complete follow-up after hospital discharge. The INSEE register of death is regularly updated as municipalities send weekly reports of death certificates of their citizens. We looked at in-hospital mortality, defined as any death occurring during a hospital stay, and one-year mortality, defined as any death reported on the INSEE register within one year of surgery. Data were anonymized before entry onto a secure internet-based electronic case record form designed specifically for our study. All data are available at https://doi.org/10.5061/dryad.tht76hf1v.

### 2.4. Outcomes

Primary outcome was pPOLOS and secondary outcomes were unplanned postoperative ICU admission, in-hospital mortality and one-year postoperative mortality. Prolonged POLOS (pPOLOS) was defined as a postoperative hospital length of stay (LOS) greater than or equal to the 75th percentile of LOS after endoscopic transsphenoidal pituitary surgery in the current cohort, including the day of discharge, irrespective of its reason. Unplanned postoperative ICU admission was defined as any postoperative ICU admission not preceded by a preoperative ICU flag. In-hospital mortality was defined as any death occurring during the hospital stay. One-year post-surgical mortality was defined as any death reported within one year of surgery.

### 2.5. Statistical Analysis

Given a small anticipated effect size with a Cohen’ f2 of 0.15, a desired statistical power of 0.8, the number of predictors included in the model (13) and a type 1 error rate of 0.05, the calculated minimal required sample size was 131 [16,17].

Continuous patient variables were summarized as the mean value and standard deviation, and categorical variables were summarized as the number and percentage of patients. Comparisons between groups were made using either the Wilcoxon test for continuous variables or Chi-Squared test for categorical variables or the log-rank test for Kaplan–Meier survival curves. We developed a descriptive logistic model (global model) that included preoperative medications and obesity, defined as a body mass index of 30 kg·m^−2^ or more (as it is an important comorbidity whose presence cannot be estimated with medications). Topical treatment classes (dermatological ATC-D class, sensory organ ATC-S class and non-classified medications ATC-V class) were not included in the analysis. Age was integrated into the model as a potential effect modifier, as it is a patient demographic known as a risk factor for prolonged postoperative length of stay in all surgical populations [18], through inclusion of an interaction term with all preoperative medications. Variable selection was then done with a backward elimination (BE) algorithm, with the Akaike Information Criterion (AIC) as a stopping criterion. This method starts with a full model that considers all the variables to be included in the model. Variables are then deleted one by one from the full model until all remaining variables in the model have a *p*-value smaller than a threshold determined by the AIC. Selection stability investigation was then performed using bootstrap resampling with replacement. One thousand resamples were drawn from the original data set and variable selection was repeated in each of the resamples. We reported bootstrap inclusion frequencies to quantify how frequently an independent variable was selected. We also reported sampling distributions of regression coefficients, the 2.5th and 97.5th percentiles of the resampled regression coefficients serving as resampling-based confidence intervals. Random forest analysis was performed as an agnostic comparator and variable importance plots were reported. All statistical analyses were done using R (R version 4.0.2, R Foundation for Statistical Computing, Vienna, Austria). The Stats package was used for regression analysis [19] (glm function for construction of the global model, step function for the AIC backward variable selection), and the randomForest package for random forest analysis [20].

## 3. Results

### 3.1. Patient Characteristics

**Figure 1:**
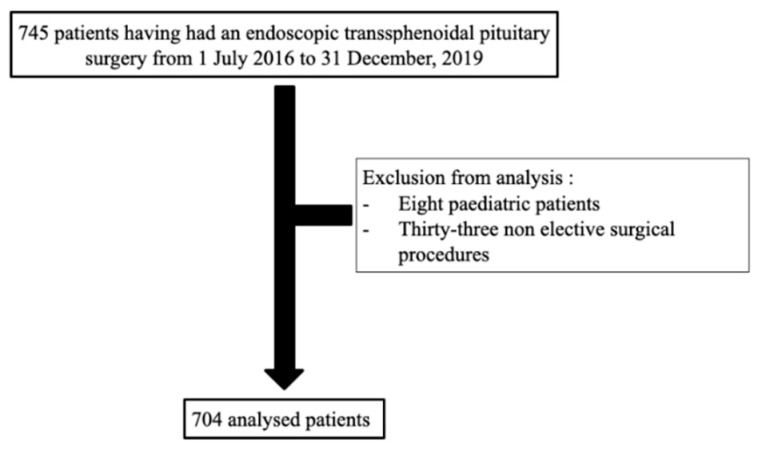
Flow diagram.

A total of 745 patients had a GHM code eligible for inclusion in the analysis. Eight pediatric patients were excluded, and thirty-three patients were excluded because their surgery was not elective. No patients were in the ICU before surgery. From the complete database, 704 patients remained for analysis after exclusion. Figure 1 shows the flow diagram.

There were no missing data in covariates included in the model, nor in the outcome variables. One hundred seventy-four patients had a prolonged postoperative length of stay. Table 1 shows preoperative patients and tumor characteristics. Median postoperative stay was 3 days and the 75th percentile of postoperative length of stay was 4 days. The extent to which the hospital stay was prolonged beyond 4 days is represented in the Supplementary Materials (Figure S1). Half of the patients with prolonged LOS (median of extended LOS) stayed for more than two extra days (total LOS 6 days) and 25% of them stayed longer than three extra days (total LOS 7 days) Fifty percent of pituitary surgery patients with a prolonged postoperative length of stay had either a cerebrospinal fluid (CSF) leak (49 patients) or diabetes insipidus (37 patients). Thirty three percent (58 patients) had no identifiable postoperative complications. The remaining 17% of patients (30 patients) had other complications. No single complication, other than CSF leak and DI, was highly represented in our population.

### 3.2. Preoperative Medications

The mean number of preoperative medications was 2.5 ± 2.6. The details of preoperative medications are available in the Supplementary Materials (Table S2). Of patients with a prolonged postoperative length of stay, 57 (33%) had thyroid replacement therapy (ATCH-03) and 48 (28%) had corticosteroid replacement therapy (ATH-02). The global model events per variable ratio (EPV global) amounted to 13.4.

### 3.3. Factors Associated with a Prolonged Postoperative Length of Stay

Table 2 reports adjusted odds ratios for prolonged postoperative length of stay in transsphenoidal hypophysectomy patients. Variables included in the model after backward elimination were obesity, age and ATC-B, ATC-H, ATC-L, ATC-N and ATC-R drugs. ATC-H drugs were the only variable significantly associated with pPOLOS, with a *p*-value < 0.001. Patients taking ATC-H drugs were at an increased risk of a prolonged postoperative length of stay; the adjusted odds ratio for every additional ATC-H drug was 1.52 (95% CI 1.22–1.90, *p*-value < 0.001).

Mean postoperative length of stay for patients taking ATC-H drugs preoperatively was 4.48 days (±5.41), compared to 3.38 days (±1.74) for those not taking ATC-H preoperatively (*p*-value = 0.002) (Table 3).

**Figure 2:**
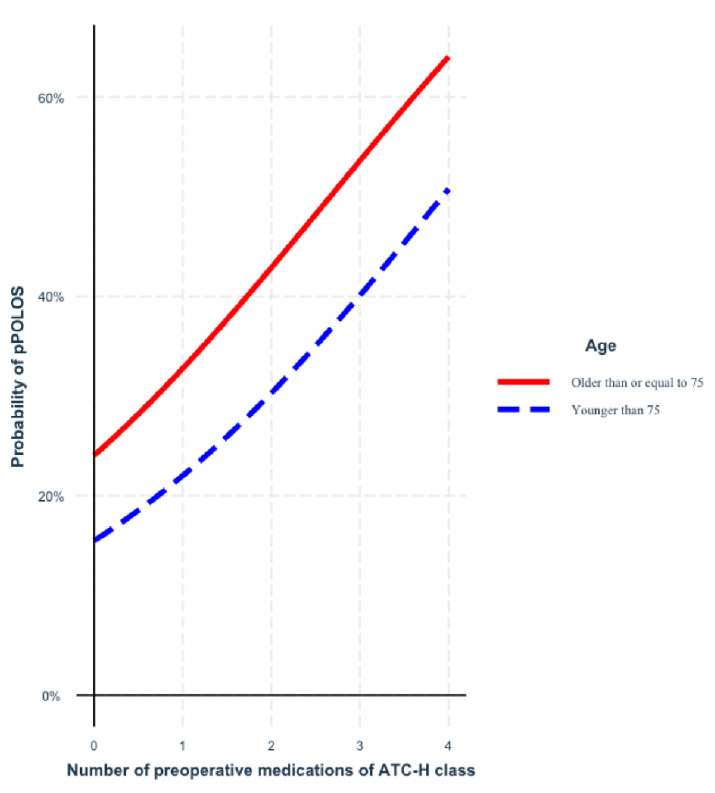
Probability of prolonged postoperative length of stay (pPOLOS) for each additional ATC-H drug, depending on patient’s age. ATC-H = ATC classification code H—systemic hormonal preparations, excluding sex hormones and insulins; pPPOLOS = prolonged postoperative length of stay.

Figure 2 shows the increased probability of prolonged postoperative length of stay for each additional ATC-H drug, irrespective of patient age. ATC-H drugs were included in 99.1% of the bootstrapped samples’ final models after backward elimination.

The distribution of odds ratios related to taking ATC-H drugs in the bootstrapped subsamples is reported in the Supplementary Materials (Figure S2). No significant association was found between preoperative ATC-H medication and type of adenoma, size of adenoma or redo surgery. No association was found between preoperative ATC-H medication and unplanned ICU admission, in-hospital mortality or postoperative complications (Table 3).

**Figure 3:**
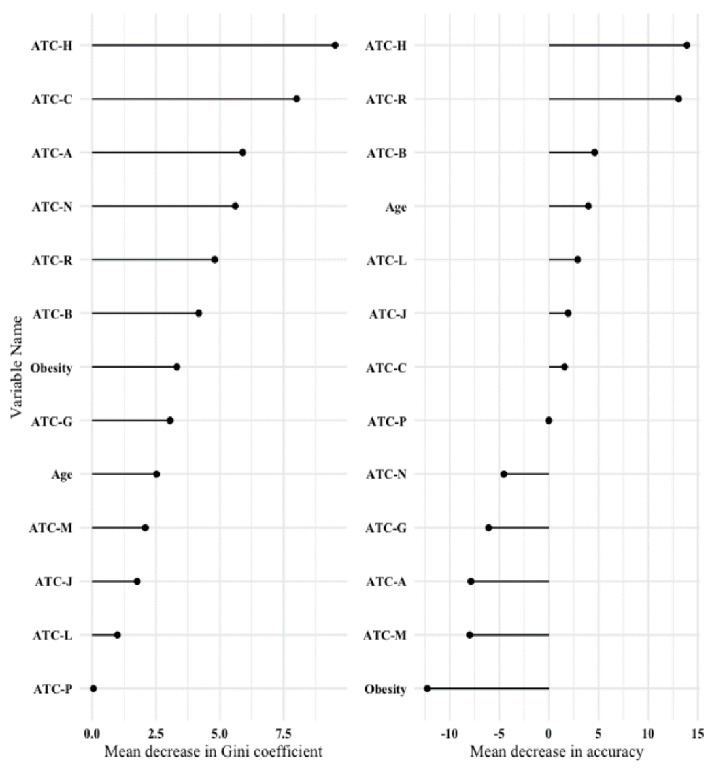
Variable importance plot after a random forest classification algorithm. Left panel: Mean decrease in Gini coefficient (a measure of how each variable contributes to the homogeneity of the nodes and leaves in the resulting random forest); Right panel: Mean decrease in accuracy (a measure of how much accuracy the model loses by excluding each variable). The higher the value of mean decrease in accuracy or mean decrease in Gini score, the higher the importance of the variable in the model. ATC-A = number of preoperative ATC classification code A medications—alimentary tract and metabolism; ATC-B = number of preoperative ATC classification code B medications—blood and blood-forming organs; ATC-C = number of preoperative ATC classification code C medications—cardiovascular system; ATC-G = number of preoperative ATC classification code G medications—genito-urinary system and sex hormones; ATC-H = number of preoperative ATC classification code H medications—systemic hormonal preparations, excluding sex hormones and insulins; ATC-J = number of preoperative ATC classification code J medications—anti-infectives for systemic use; ATC-L = number of preoperative ATC classification code L medications—antineoplastic and immunomodulating agents; ATC-M = number of preoperative ATC classification code M medications—musculo-skeletal system; ATC-N = number of preoperative ATC classification code N medications—nervous system; ATC-P = number of preoperative ATC classification code P medications—antiparasitic products, insecticides and repellents; ATC-R = number of preoperative ATC classification code R medications—respiratory system.

Three patients died within one year of surgery; all were under ATC-H medication preoperatively. The three deaths occurred on the same day as surgery, 29 days after the surgery and 39 days after the surgery, respectively. A random forest classification algorithm was applied to the data as an agnostic comparator. It also confirmed the importance of preoperative ATC-H medication on the event of prolonged postoperative length of stay. Figure 3 shows the variable importance plots.

## 4. Discussion

Preoperative treatment with systemic hormones (ATC-H drugs) is associated with an increased risk of a longer postoperative stay after transsphenoidal pituitary surgery. Postoperative CSF leakage and diabetes insipidus were the most frequently encountered complications among patients with pPOLOS. This is in accordance with the reports of Lobatto et al. in their systematic review [21].

Surgeons are at the forefront of predicting operative risk, but analyses of their subjective predictions have shown limited reliability [22,23]. Prediction scores [24,25,26] and machine learning algorithms [27] are not specific to the minimally invasive transsphenoidal hypophysectomy. Patient-centered indices have demonstrated significant predictive value for cost-related outcomes [28], but are either composed of subjective components or are too burdensome to implement clinically. As preoperative risk assessment ought to be simple [29], a medication-based risk assessment is an interesting approach. A major advantage of this approach is that it allows for risk calculation to be automated. To our knowledge, there are no studies on the association between preoperative medication and postoperative outcomes in pituitary surgery. Lobatto et al. [21] reported that age, BMI, tumor size and intraventricular extension are associated with postoperative complications after pituitary surgery. In the general surgical population, some authors have reported an association between antidepressants, anti-anxiety medications or opioids and increased POLOS [30,31], but neurosurgery represented only 5% of patients.

Blitz et al. [7] found an association between preoperative treatment and some postoperative complications in a heterogeneous population, using the analysis of a selection of 46 drugs of different ATC levels. We chose to include all systemic drugs of the same ATC level, which allowed for a more comprehensive and homogeneous analysis. Furthermore, they reported an increased risk of complications with 12 drugs, including anticonvulsant agents, aspirin and direct thrombin inhibitors especially. We did not find any association between these drugs and pPOLOS. This can partly be explained by the fact that all scheduled patients in our cohort had a preoperative consultation with the anesthesiologist, with preoperative discontinuation of drugs known to be associated with increased perioperative risk.

Medication-based approaches have been used to study one-year hospitalization [32] in non-surgical populations, mortality, unplanned hospitalization and hospital readmissions [33]. Our choice to use POLOS as an outcome measure was based on its proven ability to summarize postoperative care and costs [34] and non-clinical social criteria [35]. Twenty percent of patients with prolonged POLOS in our study population had no reported postoperative complications. This comes as no surprise, as non-medical reasons for POLOS are as frequent as 50% in the general surgery population [35]. The median length of stay in our study was 3.7 days, which is consistent with that observed in a meta-analysis by Gao et al. [36]. Few studies have characterized the preoperative risk factors of POLOS in a homogeneous neurosurgical population. Vimawala et al. [13] investigated prolonged POLOS after endoscopic transsphenoidal pituitary surgery, and found that a history of OSA with CPAP use significantly predicted pPOLOS. The medication-based approach does not allow for the identification of treated OSA patients.

Lawrence et al. [37] found chronic steroid use, a level-3 ATC drug hierarchically included in the ATC-H level-1 class, as a significant predictor of postoperative complications and increased length of stay after pituitary surgery. Their analysis did not include other medications as independent variables.

The number of ATC-H drugs is an indicator of the severity of preoperative hypopituitarism, a chronic condition with an increased mortality [38,39]. Hormonal replacement of hypopituitarism aims at correcting hypopituitarism and decreasing its morbidity. However, existing therapeutic regimens, despite continuous progress, remain unphysiological. This partly explains the persisting morbidity in adults with hypopituitarism with medical treatment [40]. Our cohort might not be sufficiently powered to detect a difference in postoperative complications explaining the increased postoperative length of stay associated with the frailty of patients with hypopituitarism despite replacement therapy. Furthermore, tumor size, a known risk factor for surgical complications, is correlated to the severity of hypopituitarism [41], and, thus, possibly, indirectly to the number of preoperative ATC-H medications. Patients with a higher number of preoperative ACT-H drugs might have a more important pressure effect from the tumor and a longer postoperative stay might reflect the more cautious postoperative monitoring chosen by the surgical team. The association between preoperative ATC-H drugs and pPOLOS could thus hypothetically be explained by unmeasured confounders, i.e., tumor extension and/or the degree of pituitary insufficiency (as defined by blood measurement of the pituitary hormones).

Our data are single-centered and retrospective. This design comes with inherent limitations of generalizability and consistency, and the model used is a descriptive one that aims at characterizing associations between preoperative medication and postoperative outcome. It does not allow us to draw conclusions about any causal relationship.

In our analysis, we chose variable selection on the change-in-estimate criterion, as it is often applied to select adjustment variables for explanatory models in epidemiological studies [42].

A major strength of our analysis is the careful inspection of the robustness of our estimations to small perturbations of the dataset, a very important and often ignored step, using bootstrap resampling [43], which is valuable to investigate and quantify model stability [44]. The ATC-H variable’s inclusion frequency was as high as 99.1%, reflecting a consistent effect. The 2.5th and 97.5th percentiles can be interpreted as limits of 95% confidence intervals obtained by resampling-based multi-model inference and further confirm the robustness of our results. We had no missing data in our covariables of interest or in our outcome variables. This is explained by the robust approach to the definition of preoperative medications using ATC classification. Age, BMI and postoperative length of stay are variables readily available in all electronic health records. Mortality data were obtained from the comprehensive national death register.

Although we did use the comprehensive ATC classification, we did not study the effects of combinations of drugs, and our data lacked the tumor extension variable and blood hormone level measurements, which could be confounders, as aforementioned.

Although the number of patients included in this analysis is one of the largest reported for transsphenoidal pituitary surgery, the low incidence of postoperative complications and the low mortality rate did not allow for reliable conclusions regarding these secondary outcomes. Finally, the generalizability of our results to patients undergoing transsphenoidal pituitary surgery in other hospitals or countries is questionable, for the methodological reasons stated above, and also because of the heterogeneity of perioperative management.

Future studies should test this medication-based approach on endoscopic pituitary surgery populations to confirm the association between preoperative ATC-H medication and POLOS and investigate cofounders not included in our analysis.

## 5. Conclusions

Patients receiving preoperative systemic hormonal preparation drugs (ATC-H class) and undergoing transsphenoidal pituitary surgery are at an increased risk for a prolonged postoperative length of stay. Clinicians should be aware of the possible vulnerability of pituitary surgery patients taking systemic hormones preoperatively and that the presence of such medications could potentially trigger modifications or at least readiness for modifications to the planned for postoperative resource utilization.

## Figures and Tables

**Table 1:** Preoperative patients’ characteristics.

Characteristics	
N = 7041	
Age	
51 (16)	
ASA-PS Score	
1	84 (12%)
2	536 (76%)
3	84 (12%)
Sex	
F	390 (55%)
M	314 (45%)
Obesity	
Non-obese	506 (72%)
Obese	198 (28%)
Type of Pituitary Tumor	
Null cell	293 (44%)
Somatotrophic	149 (22%)
Corticotrophic	137 (21%)
Gonadotrophic	28 (4.2%)
Lactotrophic	39 (5.8%)
Thyrotrophic	3 (0.4%)
Unknown	19 (2.8%)
Tumor Size	
Microadenoma	154 (23%)
Macroadenoma	508 (76%)
Unknown	6 (0.9%)
Redo surgery	74 (11%)

Data are presented as mean (SD) or number (percentage); SD = standard deviation; ASA-PS = American Society of Anesthesiology Physical Status.

**Table 2:** Adjusted odds ratios for prolonged postoperative length of stay after variable selection using a backward elimination algorithm with Akaike Information Criterion as stopping criterion.

Characteristic	OR ^1^	95% CI ^1^	*p*-Value
atcH total	1.53	1.24, 1.90	<0.001
atcLtotal	0.19	0.01, 0.96	0.112
Obesity	1.42	0.97, 2.05	0.069

^1^ OR = odds ratio; CI = confidence interval; ATC = Anatomical Therapeutic Chemical Classification System; ATC-H = ATC classification code H—systemic hormonal preparations, excluding sex hormones and insulins; ATC-L = ATC classification code L—antineoplastic and immunomodulating agents.

**Table 3:** Postoperative outcomes.

Characteristic	No Preoperative ATC-H Medication, N = 472 ^1^	Preoperative ATC-H Medication, N = 232 ^1^	*p*-Value ^2^
Postoperative length of stay (days)	3.4 (1.7)	4.5 (5.4)	0.002
Unplanned postoperative ICU	0 (0%)	2 (0.9%)	0.108
Postoperative in-hospital death	0 (0%)	1 (0.4%)	0.330
One-year death	0 (0%)	3 (1.3%)	0.035
Postoperative complications			0.364
No complication	20 (23%)	19 (27%)	
CSF leak	34 (40%)	20 (29%)	
Diabetes insipidus	21 (24%)	19 (27%)	
Surgical site bleeding	2 (2.3%)	0 (0%)	
Other	9 (10%)	12 (17%)	

^1^ Data are presented as mean (SD) or number (percentage); SD = standard deviation; ^2^ Wilcoxon rank sum test; Fisher’s exact test; ATC = Anatomical Therapeutic Chemical Classification System; ATC-H = ATC classification code H—systemic hormonal preparations, excluding sex hormones and insulins; ICU = Intensive Care Unit; CSF = cerebro-spinal fluid.

## Data Availability

As required by the French Protection Act concerning the use of anonymized hospital data, we obtained permission to access this database from the French Commission on Information Technology and Liberties (Commission Nationale de l’Informatique et des Libertés, CNIL). All anonymized data are available at https://doi.org/10.5061/dryad.tht76hf1v, publication date: 9 May 2022.

## Supplementary Materials

The following supporting information can be downloaded at: https://www.mdpi.com/article/10.3390/jcm11195829/s1. Figure S1: Extent to which the hospital stay was prolonged beyond 4 days; Figure S2: Distribution of odds ratios related to taking ATC-H drugs in the bootstrapped subsamples; Table S1: STROBE statement reporting checklist; Table S2: Details of preoperative medications.
